# Clonal relationship of synchronous head and neck cancer and esophageal cancer assessed by single nucleotide polymorphism-based loss of heterozygosity analysis

**DOI:** 10.1186/s12885-019-6394-6

**Published:** 2019-12-03

**Authors:** Somkiat Sunpaweravong, Sacarin Bunbanjerdsuk, Tanjitti Pongrujikorn, Chaiwat Naktang, Patrapim Sunpaweravong, Anupong Nitiruangjaras, Tanadech Dechaphankul, Natini Jinawath

**Affiliations:** 1 Department of Surgery, Faculty of Medicine, Prince of Songkla University, Songkhla, 90110 Thailand; 2 Program in Translational Medicine, Faculty of Medicine Ramathibodi Hospital, Mahidol University, Bangkok, 10400 Thailand; 3 Medical Genetics Center, Medical Life Sciences Institute, Department of Medical Sciences, Ministry of Public Health, Nonthaburi, Thailand; 4 National Center for Genetic Engineering and Biotechnology (BIOTEC), National Science and Technology Development Agency, Pathum Thani, Thailand; 5 Department of Internal Medicine, Faculty of Medicine, Prince of Songkla University, Songkhla, Thailand; 6 Department of Pathology, Faculty of Medicine, Prince of Songkla University, Songkhla, Thailand; 7 Department of Otolaryngology, Faculty of Medicine, Prince of Songkla University, Songkhla, Thailand; 8 Integrative Computational BioScience Center (ICBS), Mahidol University, Nakhon Prathom, Thailand

**Keywords:** Head and neck squamous cell carcinoma, Esophageal carcinoma, Synchronous, Second primary malignancy, SNP array, Loss of heterozygosity, Formalin-fixed-paraffin-embedded tissues

## Abstract

**Background:**

The prognoses of head and neck squamous cell carcinoma (HNSCC) and esophageal squamous cell carcinoma (ESCC) are poor, especially when both tumors occur at the same time. We examined the clonal relatedness of HNSCCs with synchronous ESCCs to confirm whether the second tumors were metastasis or separate second primary malignancies (SPMs) using loss of heterozygosity (LOH) analysis.

**Methods:**

Twenty-one pairs of formalin-fixed paraffin-embedded tissue from HNSCC patients with synchronous esophageal cancer were analyzed by single nucleotide polymorphism (SNP) array using the Illumina HumanCytoSNP FFPE-12 BeadChip (San Diego, CA), which contains approximately 300,000 probes. LOH was identified using Nexus Copy Number software (El Segundo, CA).

**Results:**

Comparing the LOH pattern between HNSCC and paired ESCC, we found that 20 out of 21 paired tissues had a high number of discordant LOHs (LOH identified solely in the primary HNSCC but not in synchronous ESCC at the same genomic location) and a low number of concordant LOHs (LOH at the same genomic location in both HNSCC and ESCC). Only one case fell into the undetermined category. Therefore, these 20 ESCCs were classified as SPMs or second field tumors (SFTs). Moreover, the HNSCC patients with molecularly confirmed esophageal SPM had significantly poorer survival than the other patients.

**Conclusions:**

We propose the use of a genome-wide SNP array as a tool to differentiate metastatic tumors from SPM/SFT. The SNP array offers genome-wide LOH information that earlier microsatellite analysis studies lack. The ability to accurately identify SPM should contribute to a better treatment plan and follow-up care of these patients.

## Background

Patients with head and neck squamous cell carcinoma (HNSCC) have an increased risk of developing second primary malignancies (SPMs) [1]. SPMs can be diagnosed as either synchronous (diagnosis within 6 months after an index tumor) or metachronous (diagnosis more than 6 months after the index tumor) [2]. The common locations of SPMs in head and neck cancer patients are the esophagus, lung or head and neck area [1]. Esophageal cancer is a serious malignancy due to its aggressive behavior. Although esophageal cancer is relatively uncommon in the United States, the incidence is higher in Asia [3]. The worldwide incidence of HNSCC combined with synchronous esophageal cancer varies from 2.7 to 12.5% [4–6]. In Thailand, the incidence of synchronous esophageal squamous cell carcinoma (ESCC) in HNSCC patients between 2009 and 2011 was 12.4% [7].

The molecular mechanism of SPM is still not fully understood. Field cancerization, proposed in 1953 [8], is one of the concepts that explains the development of SPMs. This concept suggests that the mucosa of the upper aerodigestive tract are repeatedly exposed to carcinogens, such as smoking and alcohol, leading to multiple areas of genetic aberrations called “fields”. These accumulated genetic alterations could eventually develop to multiple progressive cancers in the same or independent fields [9].

Currently, SPM is commonly diagnosed based on the clinical criteria proposed by Warren and Gates in 1932 [10]. It is crucial to exclude the metastasis before diagnosis of SPM due to the differences in prognoses and outcomes of disease, which lead to suitable plans of treatment [11]. In clinical practice, metastasis is diagnosed by clinicopathological examination. However, distinguishing a metastatic tumor from an independent primary cancer can be unclear in some situations [12]. Thus, molecular approaches are required to accurately make these distinctions. By comparing molecular patterns between the index tumor and the second tumor, Braakhuis et al. proposed the molecular classification of SPMs after index HNSCC as follows: 1) if the second tumor shares the same molecular alterations with the index tumor, it is defined as recurrence or metastasis; 2) if the index tumor and the second tumor share only some genetic alterations, the second tumor is classified as a second field tumor (SFT); and 3) if the genetic profiles of two tumors are unrelated, the second tumor is considered a SPM [2, 9]. The molecular patterns can also be used to evaluate the clonal relatedness of multiple tumors in the same patient, whether the tumors arise from a common clone or independent clonal origins [13, 14].

Previous studies used multiple microsatellite markers to screen for loss of heterozygosity (LOH) regions to assess the clonal relationship for differentiating between the SPM and metastasis in several cancers [15–18]. Advanced molecular techniques have been used in the studies of clonality, for example, single nucleotide polymorphism (SNP) array and next-generation sequencing [12, 19]. Although next-generation sequencing, in particular whole-genome sequencing, is increasingly used in the molecular study of clonal evolution [20], this technique is still not feasible in many labs due to the high cost and complexity of bioinformatic analysis. SNP array has been frequently used as a clinical diagnostic tool in hematological cancers [21], and its clinical use in solid tumors has gradually become more common [22]. SNP array is still practical and useful for genome-wide analysis of LOH at a high resolution [23].

In this study, we performed LOH analysis using a genome-wide SNP array in patients who developed both primary HNSCC and synchronous ESCC. To evaluate the clonal relatedness of two tumors for differentiating between SPM and metastasis, we compared the LOH patterns between primary HNSCC and synchronous esophageal cancer. Moreover, the survival rate of patients using their LOH ratio was analyzed.

## Methods

### Patients

The medical records of Songklanagarind Hospital, Prince of Songkla University, were searched for all patients diagnosed with synchronous head and neck cancer and esophageal cancer between January 2002 and December 2012. Inclusion criteria were as follows: 1) the head and neck cancers and esophageal cancers had to be squamous cell carcinoma; 2) both tumors had to be anatomically separated by normal mucosa based on clinical and pathological findings; the esophageal cancer had to be located at middle or lower esophagus to ensure a distance from the head and neck cancer of at least 10 cm; and 3) both tumors had formalin-fixed paraffin-embedded (FFPE) tissues available for subsequent analysis. Patient recruitment and sample collection were performed with protocols approved by the Institutional Review Board.

### SNP array analysis

FFPE tissues were sectioned. Archived hematoxylin and eosin-stained tissue slides were evaluated by a pathologist for the area with at least 70% tumor cells for manual macrodissection using a needle tip or scalpel. A minimum amount of 200 ng of DNA extracted from each FFPE tissue was quantified by the Qubit® 2.0 Fluorometer (ThermoFisher Scientific, Waltham, MA) and qualified using the Infinium FFPE QC Kit before being processed with the Infinium HD FFPE DNA Restoration Kit (Illumina, San Diego, CA), all according to the manufacturers’ protocols.

SNP array was performed with the HumanCytoSNP FFPE-12 v2.1 DNA Analysis BeadChip (Illumina, San Diego, CA), according to the manufacturer’s instructions. This array contains approximately 299,140 SNP markers spanning the entire genome with an average probe spacing of 72 kb. The data were analyzed with GenomeStudio Data Analysis Software v. 2011.1 (Illumina, San Diego, CA) and Nexus Copy Number v9.0 (BioDiscovery, Inc., El Segundo, CA) using the reference human genome (hg19/GRCh37).

### Analysis of LOH pattern

LOH results were obtained from the SNP array using Nexus Copy Number software’s default settings (SNP-FASST2 Segmentation Algorithm, a minimum of 3 probes per segment and a maximum contiguous probe spacing of 1000 kb). The minimum LOH length was set at 500 kb. Using the GenomicRanges packages in R, two LOHs at the same genomic region of each pair of HNSCC and ESCC were compared and defined as identical when more than 60% of the total length of both LOHs overlapped. Concordant LOH was then defined as the presence of identical LOHs at the same genomic region of both tumors. In contrast, when a unique LOH was identified only in HNSCC or ESCC, this case was defined as discordant LOH. The percentage of concordant (% concordant) or discordant (% discordant) LOH was calculated as the number of concordant or discordant LOH divided by the total number of LOH in HNSCC. The ratio between % discordant and % concordant LOH was subsequently calculated. Of note, the % concordant was initially added with 1 to avoid division by zero, and to create the 0–100 score ranges.

**Fig. 1:**
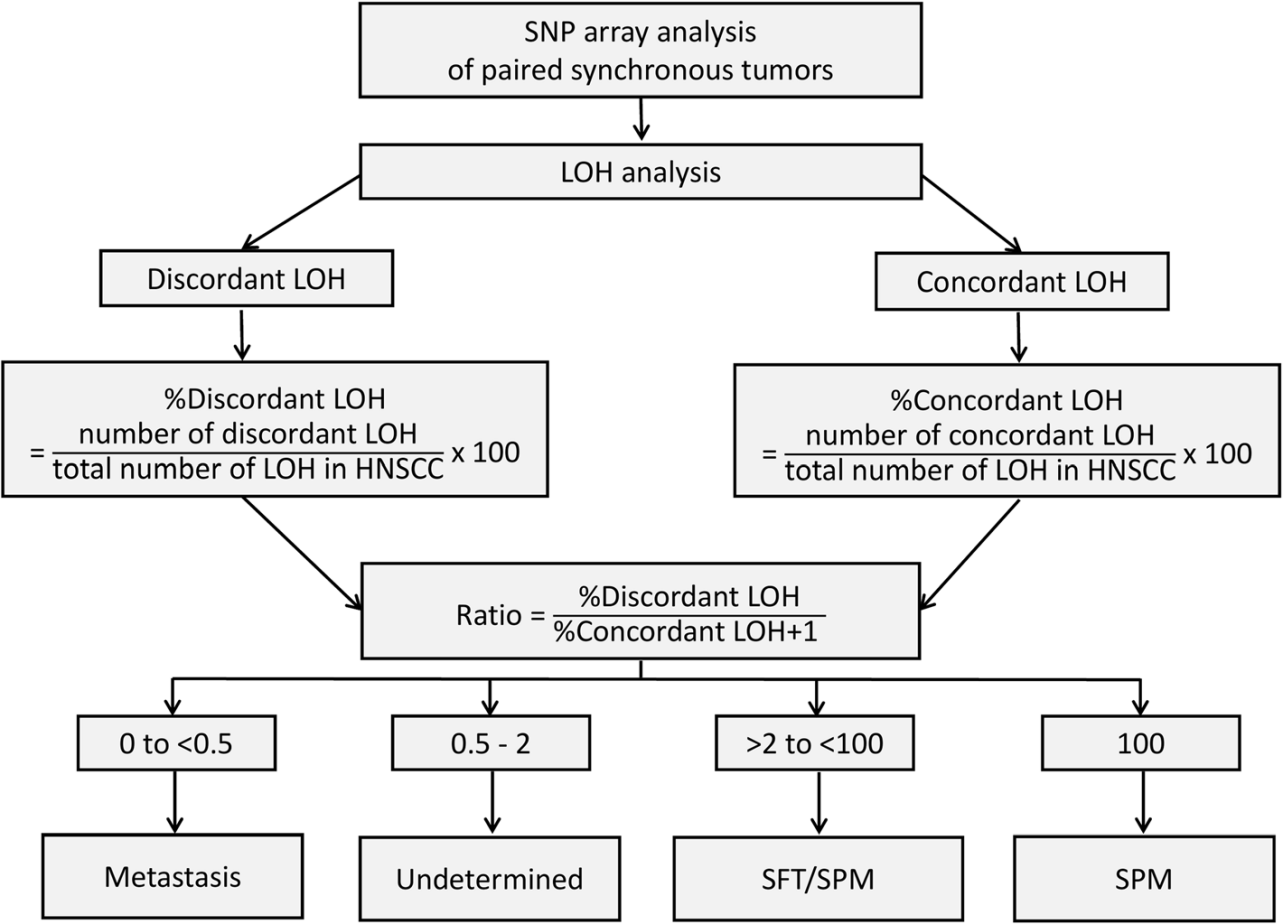
Diagram showing the classification criteria for LOH result interpretation. *SNP*, single nucleotide polymorphism; *LOH*, loss of heterozygosity; *HNSCC*, head and neck squamous cell carcinoma; *SFT*, second field tumor; *SPM*, second primary malignancy

Based on the ratio results, the second tumors were classified according to Braakhuis et al.’s proposed models [2] into four subgroups: tumors with a ratio less than 0.5 were likely to be metastatic; tumors with a ratio between 0.5 and 2 were considered undetermined; tumors with a ratio more than 2 and less than 100 could be either SFT or SPM; tumors with a ratio of 100 were considered SPM (Fig. 1).

### Statistical analysis

Statistical analyses were performed using PASW Statistics version 18.0 for Windows (SPSS Inc.) and GraphPad Prism version 6.0 for Windows (GraphPad Software). Survival analysis was performed and visualized using Kaplan-Meier curves. *P* value was generated by log-rank test.

## Results

### Patient characteristics

A total of 21 patients diagnosed with HNSCC and synchronous ESCC were included in this study. The major risk factors (i.e., smoking and alcohol) of either HNSCC or ESCC were analyzed. Patient characteristics are summarized in Table 1. The majority of patients (66.7%) were male who were active or former consumers of smoking and alcohol. Nearly half (47.6%) of the HNSCCs were located in hypopharynx. All ESCCs (100%) were located in middle and lower esophagus. The majority of HNSCCs (71.5%) were advanced stage (stage III and IV), whereas the majority of ESCCs (76.2%) were early stage (stage I and II). The difference in tumor stages implies a chronology of tumor development in which HNSCC might initially develop before ESCC, although both tumors were clinically detected at the same time.

**Table 1:** Patient characteristics

	No. (%)
Number	21
Age	
mean (years)	58.7
< 65	15 (71.4)
≥ 65	6 (28.6)
Gender	
Male	20 (95.2)
Female	1 (4.8)
Smoking	
Never	1 (4.8)
Former^a^	5 (23.8)
Active	9 (42.9)
Unknown	6 (28.6)
Alcohol consumption	
Never	0 (0)
Former^a^	3 (14.3)
Active	12 (57.1)
Unknown	6 (28.6)
Site of HNSCC	
Oropharynx	4 (19.0)
Hypopharynx	10 (47.6)
Larynx	7 (33.3)
Staging^b^ of HNSCC	
I	4 (19.0)
II	2 (9.5)
III	6 (28.6)
IV	9 (42.9)
Site of ESCC	
upper	0 (0)
middle	15 (71.4)
lower	6 (28.6)
Staging^b^ of ESCC	
I	1 (4.8)
II	15 (71.4)
III	5 (23.8)
IV	0 (0)

*HNSCC*, head and neck squamous cell carcinoma; *ESCC*, esophageal squamous cell carcinoma; ^a^Quit smoking or alcohol consumption at least 1 year before this study began; ^b^American Joint Committee on Cancer (AJCC) stage (7th ed)

### LOH analysis of paired HNSCCs and ESCCs

**Fig. 2:**
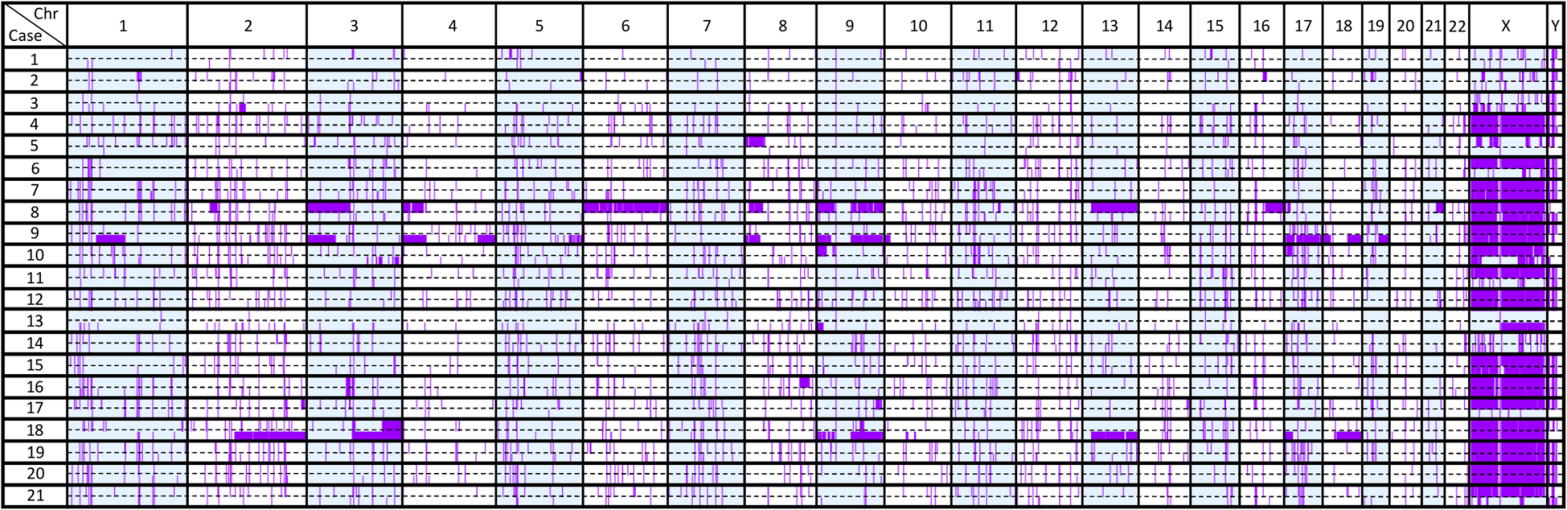
Distribution of the LOH profiles across all chromosomes in the 21 paired synchronous HNSCC and ESCC tumors. The LOH regions are shown in purple. For each case, there are two tumor samples; the upper row represents the tumor from HNSCC, while the lower row represents the matched ESCC. *Chr*, chromosome

To determine whether the tumors were two independent primary tumors or metastases, the similarity in genetic profiles were analyzed. Using SNP array, the profiles of LOHs of HNSCC and synchronous ESCC of the same patient were compared. The results represented are depicted in Fig. 2. From the LOH diagram, the majority of LOH patterns of each pair of tumors were distinguishable.

To rule out a LOH pattern that may represent tumor progression, we defined discordant LOH as a LOH identified solely in the primary HNSCC but not in the synchronous ESCC at the same genomic location. The percentages of concordant and discordant LOH were analyzed and the ratios of discordant to concordant LOH were calculated as described in Materials and Methods (Table 2). Twenty cases (95%) had a ratio of more than 2, which classified these cases as SPM or SFT. No patient was classified as metastasis (ratio < 0.5). Only one case (case 2) showed an undetermined result (ratio = 0.5–2). Moreover, 20% of the HNSCC patients with synchronous middle esophageal tumor (3 out of 15) and 50% of the patients with synchronous lower esophageal tumor (3 out of 6) showed the maximum ratio of 100, which was interpreted as molecularly confirmed SPMs.

**Table 2:** Detailed clinical characteristics and LOH analysis results of the 21 patients included in this study

No.	Sex	Age-ranges	HNSCC		ESCC		This study				Geurts et al., 2005 [16]			
			Site	Stage^a^	Site	Stage^a^	%Discordant LOH	%Concordant LOH	Ratio	Conclusion	Discordant LOH^b^	Concordant LOH	All discordant LOH	Conclusion
1	M	56–60	oropharynx	T3 N0 M0	middle	T2 N0 M0	95.45	4.55	17.2	SFT/SPM	0	1	5	SPM
2	M	61–65	oropharynx	T1 N0 M0	middle	T2 N1 M0	56.25	43.75	1.3	Undetermined	0	0	0	?
3	M	61–65	oropharynx	T2 N1 M0	middle	T2 N1 M0	100	0	100	SPM	2	0	2	SPM
4	M	36–40	larynx	T3 N3 M0	middle	T1 N0 M0	95.45	4.55	17.2	SFT/SPM	5	0	5	SPM
5	M	51–55	larynx	T1 N0 M0	middle	T2 N1 M0	92.5	7.5	10.9	SFT/SPM	4	2	12	SPM
6	M	61–65	larynx	T1 N0 M0	middle	T2 N0 M0	83.33	16.67	4.7	SFT/SPM	1	2	4	Metas?
7	M	61–65	larynx	T3 N2 M0	middle	T2 N1 M0	100	0	100	SPM	3	0	10	SPM
8	M	76–80	larynx	T3 N0 M0	middle	T2 N0 M0	93.75	6.25	12.9	SFT/SPM	4	1	7	SPM
9	M	41–45	hypopharynx	T3 N2 M0	middle	T3 N1 M0	75	25	2.9	SFT/SPM	3	2	6	SPM
10	M	41–45	hypopharynx	T4 N2 M0	middle	T3 N1 M0	92.59	7.41	11	SFT/SPM	8	0	12	SPM
11	M	51–55	hypopharynx	T2 N2 M0	middle	T2 N1 M0	100	0	100	SPM	0	0	0	?
12	M	56–60	hypopharynx	T2 N0 M0	middle	T2 N0 M0	87.1	12.9	6.3	SFT/SPM	8	0	10	SPM
13	M	61–65	hypopharynx	T3 N0 M0	middle	T3 N2 M0	81.82	18.18	4.3	SFT/SPM	4	1	12	SPM
14	F	66–70	hypopharynx	T3 N2 M0	middle	T2 N0 M0	98.25	1.75	35.7	SFT/SPM	5	1	9	SPM
15	M	81–85	hypopharynx	T1 N3 M0	middle	T2 N1 M0	93.75	6.25	12.9	SFT/SPM	0	0	1	?
16	M	71–75	oropharynx	T3 N1 M0	lower	T2 N0 M0	94.87	5.13	15.5	SFT/SPM	4	0	4	SPM
17	M	46–50	larynx	T1 N0 M0	lower	T2 N1 M0	100	0	100	SPM	7	0	8	SPM
18	M	71–75	larynx	T2 N1 M0	lower	T2 N0 M0	87.5	12.5	6.5	SFT/SPM	9	2	11	SPM
19	M	41–45	hypopharynx	T4 N2 M0	lower	T3 N2 M0	100	0	100	SPM	0	0	2	SPM?
20	M	46–50	hypopharynx	T3 N2 M0	lower	T2 N0 M0	95	5	15.8	SFT/SPM	4	0	7	SPM
21	M	51–55	hypopharynx	T2 N0 M0	lower	T2 N2 M0	100	0	100	SPM	1	0	3	SPM?

*LOH*, loss of heterozygosity; *HNSCC*, head and neck squamous cell carcinoma; *ESCC*, esophageal squamous cell carcinoma; *SFT*, second field tumor; *SPM*, second primary malignancy;?, inconclusive; *SPM?*, probably second primary malignancy; *Metas?*, probably metastasis; *M*, male; *F*, female; ^a^American Joint Committee on Cancer (AJCC) stage (7th ed); ^b^Discordant LOH not explained by tumor progression

We further analyzed our LOH data using the 25 microsatellite markers and classification method proposed by Geurts et al. [16]. Our method could determine the molecular diagnosis in cases that were not confidently defined by Geurts’ method. There was only one case with undetermined results from both methods (Table 2).

### Survival analysis

**Fig. 3:**
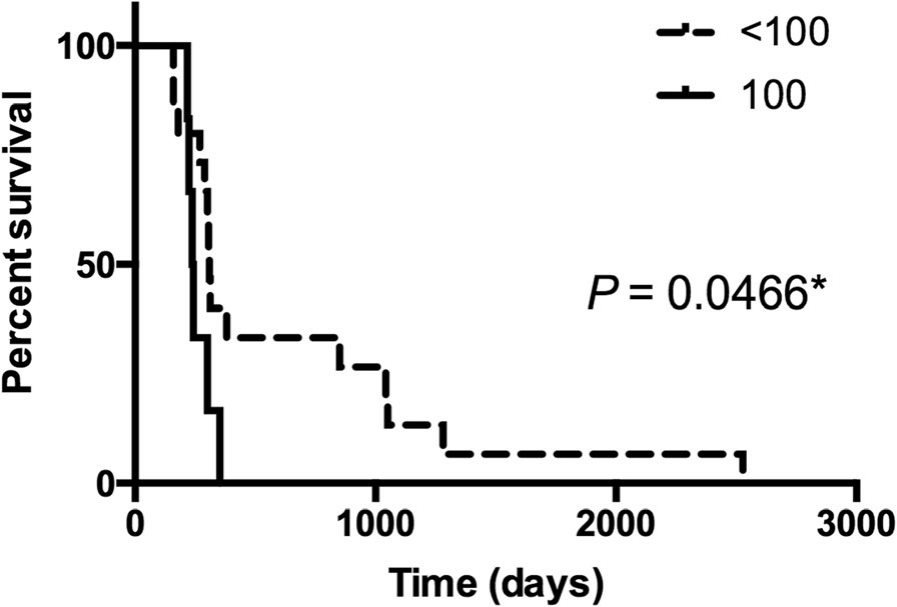
Kaplan-Meier curves showing the survival time (in days) between the HNSCC patients with molecularly confirmed esophageal SPM (ratio = 100) and the other patients (ratio < 100). ^*^Statistically significant (*P* < 0.05)

To compare the survival between HNSCC patients with molecularly confirmed esophageal SPM (ratio = 100) and the other patients (ratio < 100), Kaplan–Meier analyses were performed using time from diagnosis of the first primary HNSCC to death. The results showed that the HNSCC patients with molecularly confirmed esophageal SPM had significantly poorer survival than the other patients (log-rank test *P* = 0.0466) (Fig. 3). Furthermore, after excluding the case with molecularly undetermined clonality (ratio = 0.5–2), the Kaplan–Meier plot also showed the same pattern but without statistical significance (log-rank test *P* = 0.0738) (Additional file 1: Figure S1).

## Discussion

SPM is a leading long-term cause of mortality in patients with HNSCC and is associated with poor prognosis [24, 25]. Field cancerization is one of the major concepts that explains the mechanism of SPM development, in which multifocal tumors could originate from the same or independently genetically altered fields [2, 8]. The index tumor and metastasis are developed from the same clone. Within the field of premalignant cells, two tumors separately develop, leading to SFTs. In a case in which two tumors develop from independent fields, the second tumor is defined as SPM [2]. Therefore, the index tumor and metastasis share a strong clonal relationship. While the first tumor and SFT share some similarities in clonal patterns, the first tumor and SPM should have very few or no clonal relatedness [14].

LOH is a common genetic event in cancer development, and LOH analysis is one of the molecular techniques for studying the clonal relationship between two tumors. Many studies performed microsatellite analysis to detect LOH [15–18]. However, PCR-based LOH analysis has a limitation in these techniques use a small number of markers. SNP array is an established technology commonly used in clinical molecular cytogenetic diagnostic labs around the world for both cancers and genetic disorders [21, 23]. SNP array offers genome-wide analysis of LOH at a higher resolution, and therefore we hypothesized that it can also be used for SPM identification with a higher accuracy. Therefore, we conducted analyses using a SNP array with approximately 300,000 probes. Since there is no consensus method for analyzing the LOH profile for determining clonality, we developed classification criteria for interpretation of the LOH results using a ratio of the percentage of discordant and concordant LOH (Fig. 1). A high ratio indicates a high discordant profile, which implies a low clonal relatedness between two tumors.

In clinical practice, it is difficult to differentiate between local recurrence and SFT and also between SFT and SPM [9]. In this study, all of the selected patients were clinically diagnosed as HNSCC with synchronous esophageal SPM. The LOH analysis showed that 20 cases (95%) had the ratio of more than 2, which classified these cases as SPM or SFT. Therefore, molecular diagnosis using LOH analysis was in line with our strict clinical diagnosis, and thus confirmed the validity of using SNP array as a diagnostic tool. Moreover, our method using genome-wide SNP markers could produce more informative results than the method that used fewer numbers of markers [16]. Of note, five cases showed unclear results by Geurts’ method [16]. Using our method, these five cases could be defined as SPM/SFT. Thus, this emphasizes the benefit of using high-resolution technology such as SNP array for LOH analysis.

In a subgroup of patients, the LOH ratio was 100 (i.e. the discordant LOH was 100% and no concordant LOH was detected.) We hypothesize that the two tumors were independently developed from separate fields and had no clonal relatedness. Thus, this group of patients suffered from HNSCC with molecularly confirmed esophageal SPMs.

The synchronous esophageal cancers defined as SFT/SPM (ratio > 2 and < 100) were found in the middle and lower esophagus. In general, the distances between the head and neck area to the middle and lower esophagus regions are approximately 10 and 15 cm, respectively [26]. Our results suggest that the diameter of genetically altered field in these patients could be at least 10 cm. These findings support a previous study showing that fields with genetically altered cells can be as large as up to 7 cm in diameter [14].

A previous study showed that the survival of HNSCC patients with clinically diagnosed esophageal SPM was significantly reduced compared with HNSCC patients without SPM [27]. In this study, the survival analysis showed that HNSCC patients with molecularly confirmed esophageal SPM (ratio = 100) had significantly poorer survival than the other patients (ratio < 100) (log-rank test *P* = 0.0466). This result suggested that tumor clonality may contribute to the survival of cancer patients. A recent review on the evolutionary process on field cancerization by Curtius et al. [28] showed that clonal diversity is involved in the prognoses of many cancers. Thus, primary tumors originating from multiple different fields might be associated with poor prognosis.

To the best of our knowledge, this study is the first SNP array-based genome-wide LOH analysis and clinical correlations to differentiate the cause of HNSCC with synchronous ESCC. Our findings may indicate another important benefit to the clinical application of a SNP array test in solid tumors, which is to help guide clinical decision with regard to patients with synchronous tumors. Further study in larger HNSCC patient cohorts who have clinically-defined metastasis, local recurrent or second tumor with closer anatomical location to the first primary tumor may help clarify the usefulness of SNP array and LOH scoring system for the diagnosis of SPM in clinical practice.

## Conclusions

There is a 95% concordance between SNP array-based LOH analysis and our strict clinical diagnostic criteria for esophageal SPM, which confirmed the validity of using SNP array as a diagnostic tool. The SNP array offers genome-wide LOH information that earlier microsatellite analysis studies lack. The data generated by SNP array is also more compact and less computational intensive to analyze compared with whole exome or whole genome sequencing data, thus making it an ideal choice for clinical laboratories. The ability to accurately distinguish SPM from metastatic tumor should contribute to a better treatment plan and follow-up care of HNSCC and esophageal cancer patients.

## Supplementary information

**Additional file 1: Figure S1.** Kaplan-Meier curves showing the survival time (in days) between the HNSCC patients with molecularly confirmed esophageal SPM (ratio = 100) and the patients defined as SFT/SPM (ratio > 2, but < 100), excluding a case with undetermined result.

## Data Availability

The datasets generated during and/or analyzed during the current study are available from the corresponding author on reasonable request.

## Abbreviations

ESCC: Esophageal squamous cell carcinoma
FFPE: Formalin-fixed-paraffin-embedded
HNSCC: Head and neck squamous cell carcinoma
LOH: Loss of heterozygosity
SFTs: Second field tumors
SNP: Single nucleotide polymorphism
SPMs: Second primary malignancies
