# Supplementary figures and images for: Clonal relationship of synchronous head and neck cancer and esophageal cancer assessed by single nucleotide polymorphism-based loss of heterozygosity analysis
Source: BMC Cancer. 2019 Dec 3;19:1174. doi: 10.1186/s12885-019-6394-6 (PMC6889604; doi:10.1186/s12885-019-6394-6)

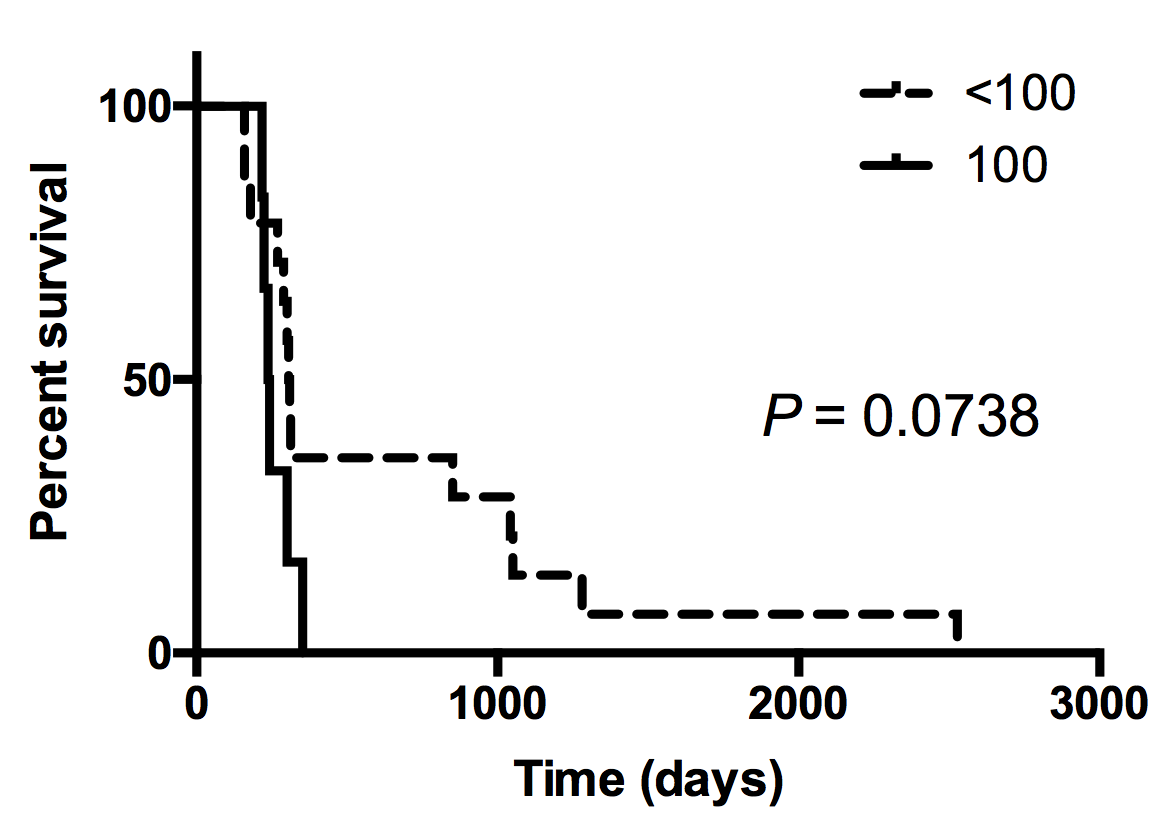

Supplement: Supplementary file 1 — Additional file 1: Figure S1. Kaplan-Meier curves showing the survival time (in days) between the HNSCC patients with molecularly confirmed esophageal SPM (ratio = 100) and the patients defined as SFT/SPM (ratio > 2, but < 100), excluding a case with undetermined result. [file 12885_2019_6394_MOESM1_ESM.tiff]
